# Clinical trajectories after preoperative traditional Chinese medicine in biologic-refractory surgery- intended acute severe ulcerative colitis: an exploratory retrospective case series

**DOI:** 10.3389/fimmu.2026.1830986

**Published:** 2026-08-24

**Authors:** Pengfei Tian, Zonglin Chen, Xin Yu, Biaojie Fang, Zhengjian Chen, Kexun Yu, Mingdian Lu

**Affiliations:** 1 Department of General Surgery, Fuyang Hospital of Anhui Medical University, Fuyang, Anhui, China; 2 Department of General Surgery, The First Affiliated Hospital of Anhui Medical University, Hefei, China

**Keywords:** acute severe ulcerative colitis, postoperative recovery, preoperative optimisation, traditional Chinese medicine, ulcerative colitis

## Abstract

**Objective:**

Acute severe ulcerative colitis (ASUC) is a life-threatening condition, and biologic-refractory patients may ultimately require surgery. The clinical role of individualised integrative management including traditional Chinese medicine (TCM) in surgery-intended ASUC remains uncertain.

**Methods:**

This single-centre retrospective exploratory case series included 39 biologic-refractory, surgery-intended ASUC patients treated between January 2020 and June 2025. Eleven accepted adjunctive preoperative TCM and 28 did not. Five selected TCM-treated patients experienced remission after integrative management including TCM and surgery was cancelled; 6 underwent surgery and were compared descriptively with 28 non-TCM surgical patients. Baseline imbalance was summarised using absolute standardised mean differences (SMDs). Continuous postoperative outcomes were reported as Hodges-Lehmann location shifts with 95% confidence intervals (CIs), and binary outcomes as risk differences with Newcombe-Wilson 95% CIs. Clinically relevant postoperative outcomes were prioritised, whilst postoperative day (POD) 1 white blood cell count (WBC) and POD3 C-reactive protein (CRP) were retained as exploratory inflammatory markers.

**Results:**

Important baseline imbalances were present in the surgical comparison cohort, including surgical scope, urinalysis, haemoglobin, haematocrit, albumin, platelet count, baseline WBC, and CRP. Thirty-day postoperative complications occurred in 10/28 (35.7%) non-TCM patients and 1/6 (16.7%) TCM surgical patients (risk difference -19.0 percentage points, 95% CI -42.0 to 23.4). The Hodges-Lehmann location shift was -4.00 days (95% CI -11.00 to 3.00) for length of stay, -3.17 mg/L (95% CI -6.15 to 0.48) for POD3 CRP, and -2.59 ×10^9^/L (95% CI -6.98 to 0.76) for POD1 WBC. No 95% CI excluded the null value. In the five selected remission cases, pre-treatment Mayo endoscopic subscore (MES) was 3 and post-treatment MES was 0 or 1; surgery was cancelled, four patients subsequently received infliximab maintenance therapy, and no subsequent surgery was recorded during 27–48 months of available follow-up.

**Conclusions:**

This case series describes heterogeneous clinical trajectories after individualised integrative management including TCM in selected biologic-refractory, surgery-intended ASUC patients. The small non-randomised cohorts, major baseline imbalance, concomitant treatment, surgical heterogeneity, and imprecise effect estimates preclude efficacy or causal inference. The observations should be considered descriptive and hypothesis-generating.

## 1 Introduction

Acute severe ulcerative colitis (ASUC) represents one of the most critical phenotypes of ulcerative colitis (UC), characterised by abrupt onset, high inflammatory burden, and frequent complications (1, 2). Although corticosteroids and rescue therapies such as cyclosporine or infliximab can salvage a subset of patients, a considerable proportion remain refractory or relapse rapidly and ultimately require surgery to control life-threatening complications such as massive haemorrhage, perforation, or toxic megacolon (3). Surgery provides rapid control of the diseased bowel but is also associated with perioperative infection, nutritional and functional impairment, and reduced quality of life related to stoma formation or staged operations (4, 5). Therefore, in the management of ASUC, two parallel objectives coexist: avoiding colectomy when safely possible and optimising perioperative status when surgery is unavoidable.

Preoperative optimisation is a major determinant of postoperative outcomes in inflammatory bowel disease (IBD) surgery (6). Existing strategies mainly include nutritional support, correction of anaemia and hypoalbuminaemia, infection control, and tailored surgical planning (7). However, these measures may be difficult to implement rapidly in biologic-refractory ASUC, where mucosal barrier disruption, innate and adaptive immune activation, microbiota dysbiosis, ongoing bleeding, and systemic catabolism coexist (8, 9).

TCM has been widely applied in chronic inflammatory gastrointestinal disorders and has been proposed to influence the gut microbiota-intestinal immunity axis, epithelial barrier integrity, and inflammatory signalling pathways (10–12). Formulations related to Shen-Ling-Bai-Zhu San and Ge-Gen-Qin-Lian Tang have been studied in UC and experimental colitis models, with reported effects on mucosal barrier restoration, inflammatory pathway regulation, and microbiota homeostasis (10, 13, 14). However, existing evidence mainly concerns mild-to-moderate UC or conservative medical management, whereas data on preoperative TCM in biologic-refractory ASUC patients already considered for surgery remain very limited (15–17).

This study was therefore designed as an exploratory retrospective case series to describe clinical trajectories after individualised integrative management including TCM in biologic-refractory, surgery-intended ASUC. We evaluated two distinct components: a descriptive postoperative comparison between TCM-treated patients who still underwent surgery and non-TCM surgical patients, and a separate remission case series of selected TCM-treated patients whose surgery was cancelled after clinical reassessment. The purpose was not to estimate treatment efficacy but to describe clinical signals, quantify uncertainty, and identify methodological requirements for future prospective studies.

## 2 Methods

### 2.1 Study design and ethics

This was a single-centre retrospective exploratory case series using human clinical data. Consecutive adult patients with ASUC who were evaluated for surgery at a tertiary referral hospital in Hefei, China, between January 2020 and June 2025 were screened. The protocol was approved by the Institutional Review Board of the First Affiliated Hospital of Anhui Medical University (PJ-2025-02-59) and conducted in accordance with the Declaration of Helsinki. Because the study was retrospective, additional informed consent for study participation was waived. Patients receiving TCM had provided routine written consent for clinical treatment. The institutional approval covered retrospective use of de-identified clinical data and endoscopic images; no identifiable patient information is presented. The study was reported in accordance with the PROCESS guideline where applicable.

### 2.2 Participants

Eligible participants were adults aged 18 years or older with UC confirmed by endoscopic and/or histopathological findings, severe disease activity according to the Modified Truelove and Witts Severity Classification, biologic-refractory disease, and documented surgery-intended status. Surgery-intended status was defined by multidisciplinary reassessment after failure of medical therapy, with a recommendation for colectomy, documented gastrointestinal surgical consultation, surgical consent, and operative scheduling before any later cancellation. The multidisciplinary process was initiated by gastroenterology or gastrointestinal surgery and included gastroenterologists, gastrointestinal surgeons, nutrition specialists, and TCM physicians when applicable. Abstracted surgical indications included corticosteroid failure, biologic failure, refractory symptoms, and, when present, massive bleeding, toxic megacolon, or perforation risk. Biologic-refractory disease included persistent active disease, loss of response, or intolerance after biologic therapy leading to surgical referral. Retrievable case-level biologic and rescue-treatment information is presented in **Supplementary Table 3**; treatment duration, trough concentration, and anti-drug antibody results were not routinely available in the retrospective records.

Patients were excluded if they had concurrent active malignancy or colorectal cancer, major comorbidities constituting an anaesthetic or surgical contraindication, uncontrolled systemic infection, a documented contraindication or severe allergy to the planned herbal treatment, or missing critical clinical variables that precluded reliable analysis.

### 2.3 Exposure assignment and analytical cohorts

Patients were classified according to whether they accepted adjunctive preoperative TCM before the planned operation. Eleven patients accepted TCM and 28 declined this option. Treatment selection was based on shared decision-making and was not randomised. Clinicians presented available management options without a protocol-mandated preference for TCM. All patients had initially accepted surgery if medical treatment remained ineffective; the five later cancellations followed clinical reassessment rather than initial refusal of surgery. Patients were generally admitted under gastroenterology and transferred to gastrointestinal surgery when an operation proceeded. No major financial barrier, pandemic-related restriction in medical access, or logistics-related delay was documented in the available records, but these factors were not prospectively measured and residual selection bias cannot be excluded.

The study comprised two analytical components. First, the surgical comparison included the 6 patients who received TCM and subsequently underwent surgery and the 28 non-TCM surgical patients. This comparison was descriptive and was not interpreted as an efficacy analysis. Second, the 5 patients who received TCM and experienced remission after integrative management such that surgery was cancelled were analysed as a separate descriptive case series and were not included in postoperative comparisons.

### 2.4 Traditional Chinese medicine intervention

The TCM exposure was an individualised component of integrative Chinese-Western medical management rather than a single standardised intervention. All 11 patients received a core prescription framework based on Shen-Ling-Bai-Zhu San combined with Ge-Gen-Qin-Lian Tang. Protocol-consistent modification categories were selected according to documented symptoms: haemostatic herbs for haematochezia; Angelicae Sinensis Radix and Paeoniae Radix Alba for abdominal pain; Aucklandiae Radix, Cyperi Rhizoma, or Citri Sarcodactylis Fructus for Qi stagnation; Massa Medicata Fermentata and Citri Reticulatae Pericarpium for impaired gastrointestinal function; Portulacae Herba, Agrimoniae Herba, and related herbs for purulent-bloody stools; and increased Astragali Radix or Codonopsis Radix for Qi deficiency. Each case-level prescription and daily herb dose is reported in **Supplementary Tables 2A** and **S2B**.

The prescriptions were prepared as decoctions by the institutional pharmacy under routine pharmacy quality-control procedures and administered twice daily for 5–7 days, with approximately 150–200 mL per dose. Case-level formulation, administration, duration, modification rationale, and adverse-event documentation are provided in **Supplementary Tables 2A** and **S2B**. Specific herb batch identifiers were not consistently retrievable from archived records, and a formal structured herb-drug interaction assessment was not documented. Accordingly, the exposure is described throughout as individualised integrative management including TCM, and observed outcomes are not attributed to a single formula or herb component.

### 2.5 Outcomes and definitions

Postoperative clinical outcomes were prioritised as exploratory endpoints, including 30-day complications, Clavien-Dindo grade ≥II and ≥III complications, readmission, reoperation, length of stay, postoperative ileus assessed using I-FEED, tolerance of solid diet within 24 hours, and time to first stool after ostomy. POD1 WBC and POD3 CRP were retained as secondary exploratory inflammatory markers because they are affected by surgical stress, corticosteroid exposure, infection, haemodilution, operative scope, and perioperative support. Other laboratory indices on POD1, POD3, and POD5 and IBDQ at admission and POD30 were reported descriptively.

For the remission cohort, outcomes were descriptive and included WBC, CRP, haemoglobin, platelet count, total Mayo score, Lichtiger score, IBDQ, endoscopic findings, subsequent treatment, relapse, subsequent surgery, and current status. Clinical remission was defined as a total Mayo score ≤1 without systemic symptoms; the endoscopic component was included. Endoscopic remission was defined as MES 0 or 1. Two endoscopists independently assessed de-identified images in a blinded manner, and comparable bowel segments were used where available. Complete paired images for all five remission cases are provided in **Supplementary Figure 2**.

### 2.6 Data collection and perioperative variables

Demographic and clinical data were extracted from electronic medical records, laboratory systems, endoscopy records, and surgical information systems by two investigators, with discrepancies adjudicated by a third investigator. Variables included demographics, disease activity, surgical indications, surgical planning and cancellation, treatment-selection factors, prior biologics and rescue therapy, corticosteroids, antibiotics, enteral and parenteral nutrition, albumin infusion, transfusion, intravenous iron, anticoagulation, antidiarrhoeal treatment, bowel preparation, infection-control measures, postoperative outcomes, and follow-up. Case-level biologic information is summarised in **Supplementary Table 3**, perioperative concomitant care in **Supplementary Table 4**, infection-screening results in **Supplementary Table 5**, and individualised TCM timelines in **Supplementary Figure 1**. Undocumented patient-level values were not imputed.

Perioperative corticosteroid exposure was summarised by drug, dose, duration, timing of the last preoperative dose, postoperative use, and cumulative hydrocortisone-equivalent dose (**Supplementary Table 1**). Patients without recorded corticosteroid exposure were assigned a cumulative dose of 0 mg only for the verified steroid variable. CRP was measured using an immunoturbidimetric assay and reported in mg/L, with a laboratory reference range of 0–6 mg/L. POD3 CRP values were verified against the original laboratory records, and no unit-conversion error was identified. The specific analyser model and exact sampling time were not consistently available.

### 2.7 Statistical analysis

All analyses were exploratory and hypothesis-generating. Continuous variables were summarised as median [interquartile range], and categorical variables as n (%). Baseline imbalance in the surgical comparison cohort was described using absolute standardised mean differences (SMDs); p values were not used to judge baseline comparability. For continuous postoperative outcomes, the effect direction was defined as TCM surgical cohort minus non-TCM cohort and reported as the Hodges-Lehmann location shift with a 95% CI derived from Wilcoxon rank-sum order statistics. Binary outcomes were reported as risk differences in percentage points with Newcombe-Wilson 95% CIs. Because the TCM surgical subgroup contained only 6 patients and major baseline imbalances were present, multivariable adjustment and propensity-score methods were not performed. Missing data were handled by complete-case analysis, no patient-level values were imputed, and no confirmatory efficacy comparison or causal inference was attempted.

## 3 Results

### 3.1 Patient flow and analytical cohorts

**Figure 1:**
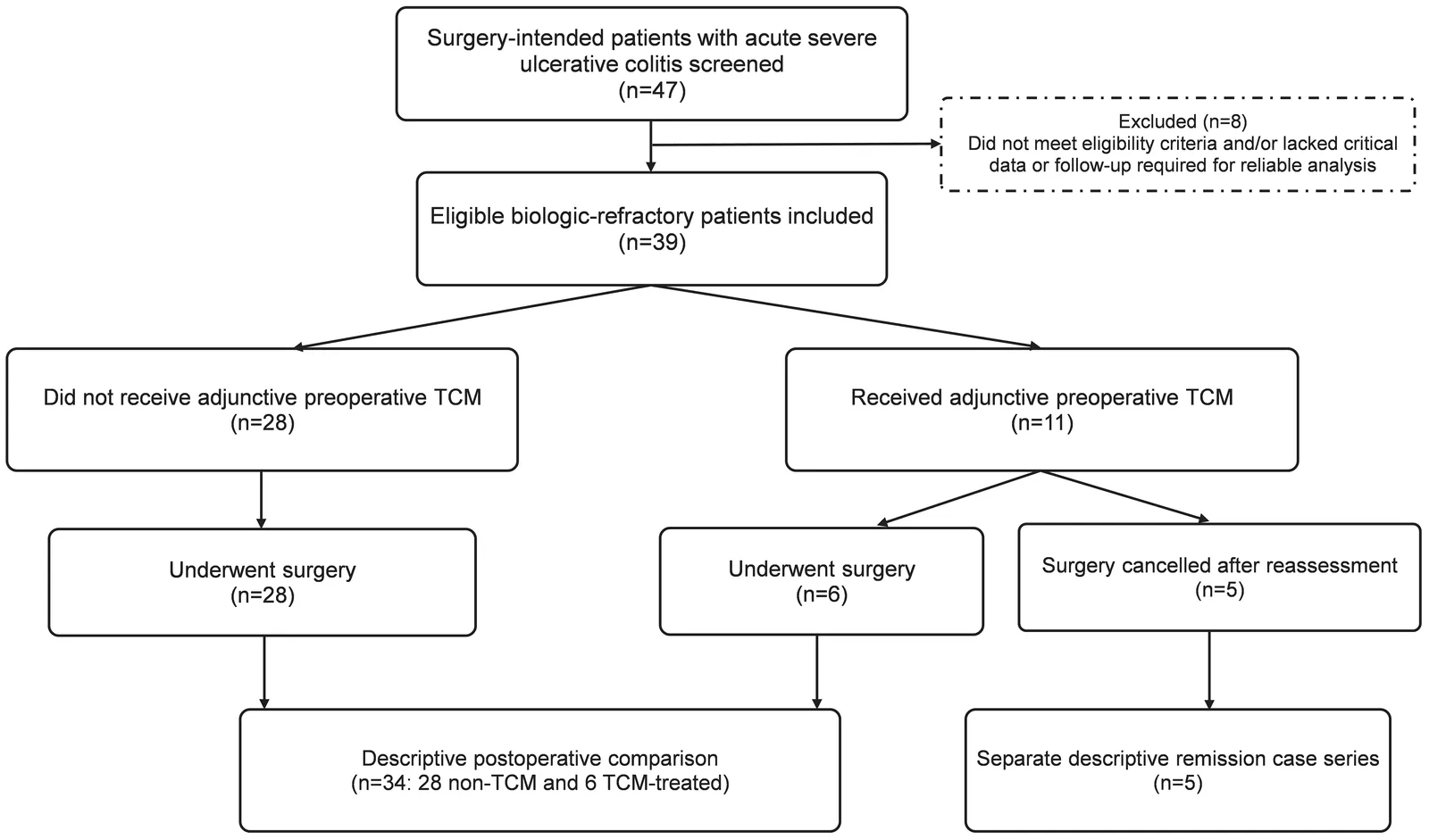
Study flow and analytical cohorts. Twenty-eight non-TCM patients and six TCM-treated patients underwent surgery and formed the descriptive postoperative comparison; five TCM-treated patients had surgery cancelled after reassessment and were described separately. ASUC, acute severe ulcerative colitis; TCM, traditional Chinese medicine.

A total of 47 surgery-intended ASUC patients were screened. Eight were excluded because they did not meet the eligibility criteria and/or lacked critical data or follow-up required for reliable analysis, leaving 39 biologic-refractory patients. Eleven accepted adjunctive TCM and 28 did not. Amongst the 11 TCM-treated patients, 5 experienced remission after integrative management and surgery was cancelled after reassessment, whereas 6 underwent surgery and entered the descriptive postoperative comparison. The study flow is shown in **Figure 1**, and individualised timelines for all 11 TCM-treated patients are shown in **Supplementary Figure 1**.

### 3.2 Baseline characteristics, surgical procedures, and corticosteroid exposure

Substantial baseline imbalance was present in the surgical comparison cohort (**Table 1**). Large absolute SMDs were observed for abnormal urinalysis (1.32), surgical scope (1.14), haematocrit (1.01), haemoglobin (0.98), albumin (0.88), AST (0.69), disease subtype (0.69), platelet count (0.68), stool examination (0.60), BMI (0.57), baseline CRP (0.55), and baseline WBC (0.55). Preoperative corticosteroid use was recorded in 11/28 (39.3%) non-TCM and 3/6 (50.0%) TCM surgical patients, and the last preoperative dose was within 24 hours in 10/28 (35.7%) and 2/6 (33.3%), respectively. These imbalances precluded interpretation as an efficacy comparison.

**Table 1:** Baseline characteristics and standardised differences in the surgical comparison cohort.

Characteristic	Non-TCM (n=28)	TCM surgical cohort (n=6)	Absolute SMD
Sex	Male: 23 (82.1%); Female: 5 (17.9%)	Male: 4 (66.7%); Female: 2 (33.3%)	0.36
Total Mayo disease-activity category	Severe: 22 (78.6%); Moderate: 6 (21.4%)	Severe: 5 (83.3%); Moderate: 1 (16.7%)	0.12
Disease subtype	Chronic relapsing type: 26 (92.9%); Initial Type: 2 (7.1%)	Chronic relapsing type: 4 (66.7%); Initial Type: 2 (33.3%)	0.69
History of abdominal operation	Yes: 3 (10.7%); No: 25 (89.3%)	Yes: 1 (16.7%); No: 5 (83.3%)	0.17
Smoking history	Yes: 2 (7.1%); No: 26 (92.9%)	Yes: 0 (0.0%); No: 6 (100.0%)	0.39
Frequency of defecation category	1: 5 (17.9%); 2: 9 (32.1%); 3: 14 (50.0%)	1: 2 (33.3%); 2: 1 (16.7%); 3: 3 (50.0%)	0.37
Haematochezia	Yes: 28 (100.0%); No: 0 (0.0%)	Yes: 6 (100.0%); No: 0 (0.0%)	0.00
Abdominal pain	Yes: 25 (89.3%); No: 3 (10.7%)	Yes: 6 (100.0%); No: 0 (0.0%)	0.49
Diarrhoea	Yes: 25 (89.3%); No: 3 (10.7%)	Yes: 5 (83.3%); No: 1 (16.7%)	0.17
Disease extent (Montreal)	E2: 9 (32.1%); E3: 19 (67.9%)	E2: 1 (16.7%); E3: 5 (83.3%)	0.37
Surgical approach	Laparoscopic: 27 (96.4%); Open: 1 (3.6%)	Laparoscopic: 5 (83.3%); Open: 1 (16.7%)	0.44
Surgical scope	subtotal colectomy: 5 (17.9%); total colectomy: 6 (21.4%); IPAA: 17 (60.7%)	subtotal colectomy: 4 (66.7%); total colectomy: 1 (16.7%); IPAA: 1 (16.7%)	1.14
Fever	Yes: 1 (3.6%); No: 27 (96.4%)	Yes: 1 (16.7%); No: 5 (83.3%)	0.44
Urinalysis	Abnormal: 8 (28.6%); Normal: 20 (71.4%)	Abnormal: 5 (83.3%); Normal: 1 (16.7%)	1.32
Stool examination	Positive: 16 (57.1%); Negative: 12 (42.9%)	Positive: 5 (83.3%); Negative: 1 (16.7%)	0.60
Age, years	54.5 [49.0–66.2]	55.5 [43.8–71.0]	0.08
BMI, kg/m²	20.63 [18.62–22.49]	18.83 [17.29–20.39]	0.57
Body temperature, °C	36.50 [36.30–36.50]	36.30 [36.23–36.45]	0.12
WBC, ×10^9^/L	5.81 [4.51–8.18]	4.72 [4.33–5.38]	0.55
Haemoglobin, g/L	102.50 [90.50–120.75]	79.00 [76.25–83.25]	0.98
Haematocrit, %	31.85 [28.15–36.58]	25.70 [24.88–28.40]	1.01
Platelet count, ×10^9^/L	280.50 [223.50–383.25]	367.50 [291.75–423.00]	0.68
Albumin, g/L	33.45 [27.05–39.52]	26.85 [24.02–28.62]	0.88
ALT, U/L	13.50 [8.75–19.00]	10.30 [7.15–15.25]	0.50
AST, U/L	19.00 [14.00–23.75]	12.00 [11.02–14.25]	0.69
CRP, mg/L	10.73 [6.80–11.38]	16.26 [11.92–19.45]	0.55
ESR, mm/h	38.00 [38.00–38.00]	38.00 [38.00–38.00]	0.15
Preoperative corticosteroid use	11 (39.3%)	3 (50.0%)	0.22
Preoperative cumulative hydrocortisone-equivalent dose, mg	0.00 [0.00–100.00]	50.00 [0.00–100.00]	0.19
Last preoperative steroid dose within 24 h	10 (35.7%)	2 (33.3%)	0.05
Postoperative corticosteroid use	12 (42.9%)	3 (50.0%)	0.14
Postoperative cumulative hydrocortisone-equivalent dose, mg	0.00 [0.00–200.00]	50.00 [0.00–175.00]	0.00

Continuous variables are shown as median [IQR] and categorical variables as n (%). Absolute SMDs quantify baseline imbalance and are not hypothesis tests. For continuous variables, SMDs were calculated using means and pooled standard deviations whilst summaries are displayed as median [IQR]. For multilevel categorical variables, the maximum absolute category-specific SMD is shown. All patients met the Modified Truelove and Witts criteria for ASUC; the moderate/severe categories shown in the Total Mayo disease-activity row were based on total Mayo scores. TCM, traditional Chinese medicine; IPAA, ileal pouch–anal anastomosis; HC, hydrocortisone.

Retrievable prior biologic and rescue-treatment information is summarised in **Supplementary Table 3**; drug concentrations, anti-drug antibody results, and reliable line-of-therapy information were not routinely available. All 34 surgical patients had documented antibiotic exposure and parenteral nutrition, whilst other supportive treatments varied and are detailed in **Supplementary Table 4**. Infection-screening results are summarised in **Supplementary Table 5**.

### 3.3 Exploratory postoperative outcomes in surgical patients

Clinically relevant postoperative outcomes and effect estimates are shown in **Table 2**. Thirty-day postoperative complications occurred in 10/28 (35.7%) non-TCM patients and 1/6 (16.7%) TCM surgical patients, corresponding to a risk difference of -19.0 percentage points (95% CI -42.0 to 23.4). Clavien-Dindo grade ≥II complications occurred in 12/28 (42.9%) and 2/6 (33.3%), respectively (risk difference -9.5 percentage points, 95% CI -39.3 to 30.6), and grade ≥III complications occurred in 3/28 (10.7%) and 0/6 (0.0%) (risk difference -10.7 percentage points, 95% CI -27.2 to 28.9). The Hodges-Lehmann location shift for length of stay was -4.00 days (95% CI -11.00 to 3.00). Readmission, reoperation, postoperative ileus, diet tolerance, and bowel-function recovery estimates were similarly imprecise, and no clinical-outcome CI excluded the null value.

**Table 2:** Descriptive postoperative outcomes and effect estimates.

Outcome	Non-TCM (n=28)	TCM surgical cohort (n=6)	Effect measure	Estimate (95% CI)
30-day postoperative complication	10/28 (35.7%)	1/6 (16.7%)	Risk difference, percentage points	-19.0 (-42.0 to 23.4)
Clavien–Dindo grade ≥II	12/28 (42.9%)	2/6 (33.3%)	Risk difference, percentage points	-9.5 (-39.3 to 30.6)
Clavien–Dindo grade ≥III	3/28 (10.7%)	0/6 (0.0%)	Risk difference, percentage points	-10.7 (-27.2 to 28.9)
Readmission within 30 days	2/28 (7.1%)	0/6 (0.0%)	Risk difference, percentage points	-7.1 (-22.6 to 32.2)
Reoperation within 30 days	1/28 (3.6%)	0/6 (0.0%)	Risk difference, percentage points	-3.6 (-17.7 to 35.6)
Postoperative ileus (I-FEED)	2/28 (7.1%)	0/6 (0.0%)	Risk difference, percentage points	-7.1 (-22.6 to 32.2)
Tolerance of solid diet within 24 h	23/28 (82.1%)	6/6 (100.0%)	Risk difference, percentage points	17.9 (-22.4 to 35.6)
Length of stay, days	26.0 [19.8–34.5]	20.0 [19.2–27.5]	Hodges-Lehmann shift	-4.00 (-11.00 to 3.00)
Time to first stool after ostomy, days	3.00 [1.00–3.00]	2.50 [1.25–3.00]	Hodges-Lehmann shift	0.00 (-1.50 to 0.00)
IBDQ at admission	115.0 [100.0–126.2]	105.0 [100.0–110.0]	Hodges-Lehmann shift	-10.00 (-25.00 to 5.00)
IBDQ at postoperative day 30	104.0 [93.2–118.8]	118.5 [110.8–121.0]	Hodges-Lehmann shift	12.50 (-5.00 to 27.00)
POD3 CRP, mg/L	5.70 [1.75–7.15]; 0.21–35.46	1.43 [1.00–1.57]; 0.21–9.02	Hodges-Lehmann shift	-3.17 (-6.15 to 0.48)
POD1 WBC, ×10^9^/L	9.50 [6.48–13.30]	6.71 [4.85–7.76]	Hodges-Lehmann shift	-2.59 (-6.98 to 0.76)
POD1 haemoglobin, g/L	94.00 [86.50–115.50]	91.50 [79.75–97.25]	Hodges-Lehmann shift	-11.00 (-29.00 to 7.00)
POD1 platelet count, ×10^9^/L	236.00 [157.50–338.00]	284.50 [267.75–311.00]	Hodges-Lehmann shift	45.50 (-51.00 to 148.00)
POD1 albumin, g/L	31.35 [26.50–34.75]	29.35 [28.75–34.38]	Hodges-Lehmann shift	0.55 (-5.10 to 5.50)
POD1 ALT, U/L	13.50 [11.00–22.75]	11.00 [9.25–12.07]	Hodges-Lehmann shift	-3.00 (-12.90 to 1.10)
POD1 AST, U/L	22.50 [15.75–28.00]	16.65 [13.82–18.50]	Hodges-Lehmann shift	-5.00 (-12.70 to 2.30)
POD3 WBC, ×10^9^/L	6.99 [4.76–10.04]	6.95 [6.22–7.81]	Hodges-Lehmann shift	-0.04 (-3.13 to 2.10)
POD3 haemoglobin, g/L	98.00 [85.00–110.00]	97.50 [83.50–107.00]	Hodges-Lehmann shift	-1.00 (-20.00 to 18.00)
POD3 platelet count, ×10^9^/L	276.00 [177.25–315.75]	253.50 [198.75–316.50]	Hodges-Lehmann shift	17.00 (-93.00 to 127.00)
POD3 albumin, g/L	30.50 [28.18–32.30]	36.65 [30.25–41.55]	Hodges-Lehmann shift	4.25 (-1.60 to 11.60)
POD3 ALT, U/L	13.00 [9.75–18.25]	13.50 [7.50–21.00]	Hodges-Lehmann shift	-1.00 (-10.00 to 6.00)
POD3 AST, U/L	17.50 [12.75–27.75]	19.00 [13.25–24.75]	Hodges-Lehmann shift	-1.00 (-10.00 to 7.50)
POD5 WBC, ×10^9^/L	6.42 [6.05–6.66]	6.18 [5.25–6.65]	Hodges-Lehmann shift	-0.03 (-1.85 to 1.74)
POD5 haemoglobin, g/L	88.00 [87.25–90.50]	86.50 [84.25–109.00]	Hodges-Lehmann shift	0.00 (-7.00 to 28.00)
POD5 platelet count, ×10^9^/L	243.50 [223.50–273.25]	240.25 [204.00–260.38]	Hodges-Lehmann shift	-6.50 (-101.50 to 41.50)
POD5 albumin, g/L	30.80 [29.35–30.80]	32.30 [30.88–35.75]	Hodges-Lehmann shift	2.65 (0.00 to 6.40)
POD5 ALT, U/L	17.00 [16.25–17.00]	15.50 [13.25–33.50]	Hodges-Lehmann shift	0.00 (-6.00 to 22.00)
POD5 AST, U/L	19.00 [19.00–19.75]	18.50 [17.25–31.00]	Hodges-Lehmann shift	0.00 (-7.00 to 16.00)

Continuous outcomes are shown as median [IQR] and reported as Hodges-Lehmann location shifts with 95% CIs from Wilcoxon rank-sum order statistics. Binary outcomes are shown as n/N (%) and reported as risk differences in percentage points with Newcombe-Wilson 95% CIs. The effect direction is TCM surgical cohort minus non-TCM cohort. No 95% CI excluded the null value. Estimates are unadjusted, descriptive, and not evidence of efficacy. CRP, C-reactive protein; WBC, white blood cell count; POD, postoperative day; IBDQ, Inflammatory Bowel Disease Questionnaire; POI, postoperative ileus.

POD3 CRP was 5.70 [1.75–7.15] mg/L (range, 0.21–35.46) in the non-TCM group and 1.43 [1.00–1.57] mg/L (range, 0.21–9.02) in the TCM surgical cohort. The Hodges–Lehmann location shift was −3.17 mg/L (95% CI, −6.15 to 0.48). POD1 WBC was 9.50 [6.48–13.30] versus 6.71 [4.85–7.76] ×10^9^/L, with a location shift of −2.59 ×10^9^/L (95% CI, −6.98 to 0.76). These intervals included the null and should not be interpreted as evidence of a treatment effect. Verification against the original laboratory records confirmed that CRP was reported in mg/L by immunoturbidimetry and identified no unit-conversion error. No postoperative outcome had a 95% CI excluding zero.

### 3.4 Remission case series: endoscopic, laboratory, clinical changes and follow-up

**Figure 2:**
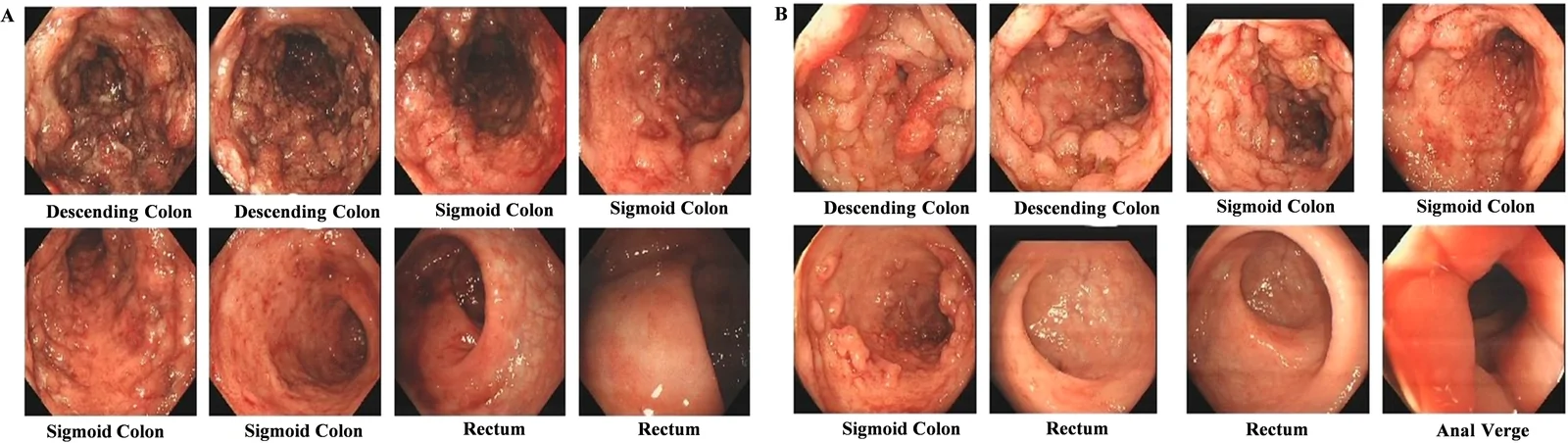
Representative colonoscopic comparison from TCM-R1. **(A)** shows pre-treatment images from the descending colon, sigmoid colon, and rectum; **(B)** shows post-treatment images from corresponding segments and the anal verge. Complete paired images for all five remission cases, with MES annotations, are provided in **Supplementary Figure 2**.

Amongst the 11 patients who received preoperative TCM, 5 selected patients experienced clinical remission after integrative management including TCM, and surgery was cancelled after reassessment. Pre-treatment MES was 3 in all five cases; post-treatment MES was 1 in TCM-R1, TCM-R3, TCM-R4, and TCM-R5 and 0 in TCM-R2. Representative images from TCM-R1 are shown in **Figure 2**, and complete paired pre- and post-treatment images from all five cases are provided in **Supplementary Figure 2**. The identifiers TCM-R1 to TCM-R5 are used consistently in **Tables 3**, **4** and **Supplementary Figure 2**. Comparable or near-comparable bowel segments were used where available, and the images were independently reviewed in a blinded manner by two endoscopists.

**Table 3:** Clinical and laboratory indices before and after integrative management in the five remission cases.

Case	Index	Pre-TCM	Post-TCM
TCM-R1	WBC (×10^9/L)	9.14	5.75
	Hb (g/L)	98	82
	PLT (×10^9/L)	394	314
	CRP (mg/L)	9.46	0.7
	Mayo score	11	0
	Lichtiger score	14	0
	IBDQ	87	196
TCM-R2	WBC (×10^9/L)	11.97	8.69
	Hb (g/L)	65	83
	PLT (×10^9/L)	357	271
	CRP (mg/L)	62.02	1.5
	Mayo score	12	0
	Lichtiger score	16	0
	IBDQ	80	210
TCM-R3	WBC (×10^9/L)	9.76	7.54
	Hb (g/L)	84	86
	PLT (×10^9/L)	114	244
	CRP (mg/L)	146.3	14
	Mayo score	10	0
	Lichtiger score	15	1
	IBDQ	94	196
TCM-R4	WBC (×10^9/L)	8.95	4.31
	Hb (g/L)	102	110
	PLT (×10^9/L)	158	172
	CRP (mg/L)	15.6	2.3
	Mayo score	11	1
	Lichtiger score	14	0
	IBDQ	75	207
TCM-R5	WBC (×10^9/L)	10.42	5.68
	Hb (g/L)	123	125
	PLT (×10^9/L)	265	246
	CRP (mg/L)	10.6	0.5
	Mayo score	9	0
	Lichtiger score	14	0
	IBDQ	94	212

The Mayo values in this table are total Mayo scores: ≥9 severe, 6–8 moderate, and ≤1 defined clinical remission in this study. Endoscopic remission was defined as MES 0 or 1. Lichtiger score: ≥10 severe, 6–9 moderate, and ≤3 remission-level disease activity. IBDQ ≥170 indicates near-normal quality of life.

**Table 4:** Follow-up of five patients whose surgery was cancelled after reassessment.

Patient ID	Follow-up duration	Relapse	Subsequent therapy	Subsequent surgery	Current status
TCM-R1	48 months	No documented relapse	Infliximab maintenance therapy	No documented surgery	Clinical remission
TCM-R2	27 months	No documented relapse	No documented biologic maintenance	No documented surgery	Endoscopic mucosal healing
TCM-R3	32 months	No documented relapse	Infliximab maintenance therapy	No documented surgery	Clinical remission
TCM-R4	38 months	No documented relapse	Infliximab maintenance therapy	No documented surgery	Clinical remission
TCM-R5	45 months	No documented relapse	Infliximab maintenance therapy	No documented surgery	Clinical remission

Follow-up data were extracted from available medical records.

Case-level laboratory and clinical trajectories are shown in **Table 3**. Across the five cases, median WBC changed from 9.76 to 5.75 ×10^9^/L and median CRP from 15.6 to 1.5 mg/L; total Mayo score decreased from 10.6 to 0.2, Lichtiger score from 14.6 to 0.2, and IBDQ increased from 86.0 to 204.2. Available follow-up ranged from 27 to 48 months, with no subsequent surgery recorded (**Table 4**). Four patients received subsequent infliximab maintenance therapy and one underwent routine follow-up without documented biologic maintenance. Therefore, the long-term surgery-free course cannot be attributed to TCM and is described as an outcome after integrative management.

## 4 Discussion

This exploratory retrospective case series generated two descriptive observations in biologic-refractory, surgery-intended ASUC. First, postoperative clinical outcomes and inflammatory markers showed numerically different point estimates between surgical cohorts, but every 95% CI included the null and the estimates were highly imprecise. Second, five selected patients experienced remission after integrative management including TCM and had surgery cancelled, but four subsequently received infliximab maintenance therapy. These trajectories are clinically informative but do not constitute evidence of TCM efficacy.

Clinical endpoints are more informative than isolated inflammatory markers in this setting. Thirty-day complications, Clavien–Dindo grades, readmission, reoperation, and length of stay were therefore prioritised in the analysis. POD1 WBC remains strongly influenced by surgical stress, corticosteroids, infection, and haemodilution. POD3 CRP is more interpretable but is affected by operative scope, infection, nutritional support, and transfusion. The Hodges–Lehmann estimates for POD1 WBC and POD3 CRP had 95% CIs spanning zero, so neither marker supports a between-group treatment effect.

Baseline imbalance and treatment selection are central limitations. Large SMDs were present for surgical scope, urinalysis, haematological and nutritional variables, platelet count, baseline inflammatory markers, and disease subtype. TCM use depended on patient choice rather than random allocation: 11 patients accepted and 28 declined. Although the available records did not identify major financial, pandemic-access, or logistics barriers, these factors were not prospectively measured. Disease trajectory, patient preference, clinical stability, clinician judgement, department transfer, and unrecorded social factors could all influence both TCM selection and surgery cancellation.

The biological rationale for TCM in ASUC remains plausible but unproven. ASUC involves epithelial barrier disruption, mucosal immune activation, microbiota dysbiosis, inflammatory signalling, bleeding, malnutrition, and systemic catabolism (2, 9). Related formulations have been reported to influence barrier integrity, microbiota composition, and inflammatory pathways (10, 11, 13, 18–20). However, the present study did not include mechanistic endpoints such as faecal calprotectin, cytokines, immune-cell profiling, microbiome sequencing, or tissue barrier markers; mechanistic explanations therefore remain speculative.

The individualised nature of the exposure is clinically authentic but scientifically limiting (21–24). The fixed core framework, modification rules, daily herb doses, administration, and case-level prescriptions are reported in **Supplementary Tables 2A** and **S2B**. Nevertheless, herbs, doses, treatment duration, and concomitant therapies varied. Specific herb batch identifiers and formal herb-drug interaction assessments were not consistently retrievable. The exposure should therefore be understood as individualised integrative Chinese-Western medical management including TCM, not as a reproducible single-formula intervention.

Additional limitations include the retrospective single-centre design, the 6-patient TCM surgical subgroup, absence of an *a priori* sample-size calculation, complete-case analysis, and inability to perform stable multivariable or propensity-score adjustment. Prior biologic dose, timing, efficacy reassessment, trough concentration, anti-drug antibody data, and line-of-therapy information were incomplete. Infection screening was not protocolised, and pathogen-directed management was not consistently available. The exact timing of some supportive treatments and the archived CRP analyser model were not consistently available. Antibiotics, nutrition, albumin, transfusion, corticosteroids, infection-control measures, and surgical procedure heterogeneity may confound postoperative outcomes. The remission series lacked a concurrent control, and spontaneous fluctuation, regression to the mean, delayed effects of prior rescue therapy, and infliximab maintenance cannot be separated from the observed course.

Despite these limitations, the study addresses a clinically difficult and under-reported population already being considered for colectomy. This study reports SMDs for baseline imbalance, effect estimates with 95% CIs, clinically relevant postoperative endpoints, verified CRP units and assay method, case-level corticosteroid and supportive-care data, documented biologic exposure, infection-screening results, individualised timelines, complete TCM prescriptions, and paired endoscopic images for all five remission cases. These materials improve transparency and may support the design of prospective multicentre studies with standardised eligibility, intervention, co-treatment, infection-screening, and outcome protocols.

## 5 Conclusion

In this exploratory retrospective case series, selected biologic-refractory, surgery-intended ASUC patients had heterogeneous clinical trajectories after individualised integrative management including TCM. Postoperative effect estimates were imprecise and all 95% CIs included the null. Five selected patients experienced clinical and endoscopic remission and surgery cancellation, but subsequent maintenance therapy and the absence of a concurrent control prevent attribution to TCM. The findings are descriptive and hypothesis-generating and require prospective confirmation.

## Data Availability

The raw data supporting the conclusions of this article will be made available by the authors, without undue reservation.
