# Supplementary material for: Clinical trajectories after preoperative traditional Chinese medicine in biologic-refractory surgery- intended acute severe ulcerative colitis: an exploratory retrospective case series
Source: Front Immunol. 2026 Aug 24;17:1830986. doi: 10.3389/fimmu.2026.1830986 (PMC13547308; doi:10.3389/fimmu.2026.1830986)
Supplement: Supplementary file 1 [file Supplementaryfile1.docx]

**Supplementary File S1. PROCESS Checklist for Surgical Case Series Reporting**

Manuscript title: Clinical Trajectories After Preoperative Traditional Chinese Medicine in Biologic-Refractory Surgery-Intended Acute Severe Ulcerative Colitis: An Exploratory Retrospective Case Series

| **PROCESS domain / item** | **Reported location in manuscript** | **Compliance / notes** |
| --- | --- | --- |
| Title | Title page | Reported. The title identifies the study as an exploratory retrospective case series and specifies the clinical context: biologic-refractory, surgery-intended ASUC. |
| Keywords | Title page / Keywords | Reported. Keywords include ulcerative colitis, traditional Chinese medicine, preoperative optimisation, postoperative recovery, and acute severe ulcerative colitis. |
| Abstract | Abstract: Objective, Methods, Results, Conclusions | Reported. The abstract states the study design, cohort size, analytical components, exploratory inflammatory markers, key results, follow-up, and limitations regarding causal inference. |
| Introduction: background and rationale | Introduction | Reported. The manuscript describes ASUC severity, surgical need, preoperative optimisation, and the uncertainty regarding preoperative TCM in biologic-refractory ASUC. |
| Introduction: objective / research question | End of Introduction | Reported. The objective is to describe clinical trajectories after individualised integrative management including TCM and to identify methodological requirements for future prospective studies. |
| Study design | Methods 2.1 and 2.3 | Reported. Single-centre retrospective exploratory case series with two analytical components: exploratory surgical comparison and descriptive remission case series. |
| Setting and timeframe | Methods 2.1 | Reported. Tertiary referral hospital in Hefei, China; January 2020 to June 2025. |
| Ethics and consent | Methods 2.1; Ethics approval and consent to participate; Consent for publication | Reported. Institutional ethics approval is stated. Retrospective consent waiver and de-identification of endoscopic images are described. |
| Participants: eligibility criteria | Methods 2.2 | Reported. Inclusion and exclusion criteria are described, including adult ASUC, biologic-refractory disease, surgery-intended status, and missing critical variables. |
| Exposure / intervention details | Methods 2.3 and 2.4; Supplementary Tables S2A and S2B | Reported. TCM exposure assignment, individualised regimen principles, core-formula framework, administration, duration, and detailed prescriptions are provided. |
| Comparator / analytical cohorts | Methods 2.3; Results 3.1; Figure 1 | Reported. Non-TCM surgical cohort, TCM surgical cohort, and TCM remission cohort are defined separately. |
| Outcome measures and definitions | Methods 2.5 | Reported. Clinically relevant postoperative outcomes are prioritised; POD1 WBC and POD3 CRP are retained as exploratory inflammatory markers. Total Mayo score and MES-based remission definitions are specified. |
| Perioperative variables and confounders | Methods 2.6; Results 3.2; Table 1; Supplementary Table S1 | Reported. Corticosteroids, antibiotics, enteral and parenteral nutrition, albumin, transfusion, intravenous iron, anticoagulation, antidiarrhoeal therapy, infection control, and bowel preparation are reported in the manuscript and Supplementary Tables S1 and S4. |
| Data extraction and reliability | Methods 2.6 | Reported. Data were extracted from electronic medical records and surgical systems by two investigators with third-investigator adjudication. |
| Statistical analysis | Methods 2.7 | Reported. Analyses are exploratory. Baseline imbalance is reported using absolute standardised mean differences. Continuous postoperative outcomes use Hodges-Lehmann location shifts with 95% CIs, and binary outcomes use risk differences with Newcombe-Wilson 95% CIs. No multivariable or propensity-score adjustment was attempted because of the small subgroup and major imbalance. |
| Patient flow | Results 3.1; Figure 1 | Reported. Screening, exclusion, TCM assignment, surgery, and remission cohorts are shown. |
| Baseline characteristics | Results 3.2; Table 1 | Reported. Demographic, disease, surgical, laboratory, and corticosteroid variables are summarised, with baseline imbalance acknowledged. |
| Postoperative outcomes | Results 3.3; Table 2 | Reported. Clinically relevant complications, Clavien-Dindo grades, readmission, reoperation, length of stay, postoperative ileus, diet tolerance, and inflammatory markers are presented with effect estimates and 95% CIs. No confirmatory efficacy inference is made. |
| Remission case series outcomes | Results 3.4; Figure 2; Table 3; Table 4 | Reported. Endoscopic, laboratory, clinical, IBDQ, subsequent therapy, documented relapse, documented surgery, and follow-up status are summarised. Table 4 and Supplementary Figure S2 use the identifiers TCM-R1 to TCM-R5. |
| Adverse events / safety | Methods 2.4; Supplementary Table S2A; Discussion | Partially reported. No TCM-related adverse events were documented in available records; retrospective documentation limits complete safety ascertainment. |
| Discussion: key findings | Discussion | Reported. The manuscript summarizes two main clinical observations and frames them as hypothesis-generating signals rather than evidence of efficacy. |
| Discussion: strengths and limitations | Discussion | Reported. Limitations include retrospective single-centre design, small subgroup size, baseline imbalance, non-randomised selection, co-treatment confounding, surgical and regimen heterogeneity, incomplete biologic and infection documentation, and lack of mechanistic endpoints. |
| Discussion: interpretation and future directions | Discussion; Conclusion | Reported. The manuscript recommends prospective multicentre studies with standardised eligibility, intervention, co-treatment, infection-screening, safety, and outcome protocols. |
| Funding | Funding section | Reported. Funding sources and lack of funder role are stated. |
| Data availability | Data availability section | Reported. Deidentified participant data and protocol availability are described as subject to institutional approval and data protection requirements. |
| Competing interests | Competing interests section | Reported. No competing interests declared. |
| Reporting statement | Reporting statement | Reported. The manuscript states that the study is an exploratory retrospective case series and identifies this completed PROCESS checklist as Supplementary File S1. |

*Checklist basis: PROCESS reporting guidance for surgical case series. The checklist is used here as a reporting aid because the present study is a retrospective exploratory case series with a surgical component.* *This checklist was completed for the present exploratory retrospective case series. Items were reported where applicable, and components not applicable to this retrospective study were marked accordingly.*
