# Supplementary material for: Clinical trajectories after preoperative traditional Chinese medicine in biologic-refractory surgery- intended acute severe ulcerative colitis: an exploratory retrospective case series
Source: Front Immunol. 2026 Aug 24;17:1830986. doi: 10.3389/fimmu.2026.1830986 (PMC13547308; doi:10.3389/fimmu.2026.1830986)
Supplement: Supplementary file 2 [file Table1.docx]

**Supplementary Table S1. Case-level perioperative corticosteroid exposure**

| **Case** | **TCM** | **Disease activity** | **Preoperative corticosteroid exposure** | **Postoperative corticosteroid exposure** |
| --- | --- | --- | --- | --- |
| 1 | No | Severe | Prednisone acetate; Prednisone acetate 20 mg bid × 4 d; total HC-equivalent dose 640 mg; last dose before surgery 1 d; within 24 h: Yes | Prednisone acetate; Prednisone acetate 10 mg bid × 9 d; total HC-equivalent dose 720 mg |
| 2 | No | Moderate | No corticosteroid exposure | No corticosteroid exposure |
| 3 | No | Severe | No corticosteroid exposure | Hydrocortisone; Hydrocortisone 25 mg qd × 2 d; total HC-equivalent dose 50 mg |
| 4 | No | Severe | No corticosteroid exposure | No corticosteroid exposure |
| 5 | No | Moderate | No corticosteroid exposure | No corticosteroid exposure |
| 6 | No | Severe | No corticosteroid exposure | No corticosteroid exposure |
| 7 | No | Severe | No corticosteroid exposure | No corticosteroid exposure |
| 8 | No | Severe | Hydrocortisone; Hydrocortisone 50 mg qd × 3 d; total HC-equivalent dose 150 mg; last dose before surgery 1 d; within 24 h: Yes | Hydrocortisone; Hydrocortisone 50 mg qd × 10 d; total HC-equivalent dose 500 mg |
| 9 | No | Severe | No corticosteroid exposure | No corticosteroid exposure |
| 10 | No | Severe | No corticosteroid exposure | Hydrocortisone sodium succinate; Hydrocortisone sodium succinate 100 mg qd × 3 d; total HC-equivalent dose 300 mg |
| 11 | No | Moderate | No corticosteroid exposure | No corticosteroid exposure |
| 12 | No | Moderate | No corticosteroid exposure | No corticosteroid exposure |
| 13 | No | Severe | No corticosteroid exposure | No corticosteroid exposure |
| 14 | No | Moderate | No corticosteroid exposure | No corticosteroid exposure |
| 15 | No | Severe | Hydrocortisone sodium succinate; Hydrocortisone sodium succinate 100 mg qd × 2 d; total HC-equivalent dose 200 mg; last dose before surgery 2 d; within 24 h: No | No corticosteroid exposure |
| 16 | No | Severe | Hydrocortisone sodium succinate; Hydrocortisone sodium succinate 100 mg qd × 2 d; total HC-equivalent dose 200 mg; last dose before surgery 1 d; within 24 h: Yes | Hydrocortisone sodium succinate; Hydrocortisone sodium succinate 100 mg qd × 2 d; total HC-equivalent dose 200 mg |
| 17 | No | Severe | Hydrocortisone sodium succinate; Hydrocortisone sodium succinate 100 mg qd × 1 d; total HC-equivalent dose 100 mg; last dose before surgery 1 d; within 24 h: Yes | No corticosteroid exposure |
| 18 | No | Severe | No corticosteroid exposure | No corticosteroid exposure |
| 19 | No | Severe | Hydrocortisone sodium succinate; Hydrocortisone sodium succinate 100 mg qd × 1 d; total HC-equivalent dose 100 mg; last dose before surgery 1 d; within 24 h: Yes | Hydrocortisone sodium succinate; Hydrocortisone sodium succinate 100 mg qod × 3 d; total HC-equivalent dose 150 mg |
| 20 | No | Severe | Hydrocortisone sodium succinate; Hydrocortisone sodium succinate 200 mg qd × 4 d; total HC-equivalent dose 800 mg; last dose before surgery 1 d; within 24 h: Yes | Hydrocortisone sodium succinate; Hydrocortisone sodium succinate 100 mg bid × 5 d; total HC-equivalent dose 1000 mg |
| 21 | No | Moderate | No corticosteroid exposure | Hydrocortisone sodium succinate; Hydrocortisone sodium succinate 100 mg qd × 4 d; total HC-equivalent dose 400 mg |
| 22 | Yes | Severe | Hydrocortisone sodium succinate; Hydrocortisone sodium succinate 100 mg qd × 1 d; total HC-equivalent dose 100 mg; last dose before surgery 1 d; within 24 h: Yes | Hydrocortisone sodium succinate; Hydrocortisone sodium succinate 100 mg qd × 2 d; total HC-equivalent dose 200 mg |
| 23 | Yes | Severe | Methylprednisolone sodium succinate; Methylprednisolone sodium succinate 40 mg qd × 3 d; total HC-equivalent dose 600 mg; last dose before surgery 2 d; within 24 h: No | Hydrocortisone sodium succinate; Hydrocortisone sodium succinate 200 mg qd × 3 d; total HC-equivalent dose 600 mg |
| 24 | No | Severe | Hydrocortisone sodium succinate; Hydrocortisone sodium succinate 100 mg qd × 1 d; total HC-equivalent dose 100 mg; last dose before surgery 1 d; within 24 h: Yes | Hydrocortisone sodium succinate; Hydrocortisone sodium succinate 100 mg qd × 2 d; total HC-equivalent dose 200 mg |
| 25 | No | Severe | No corticosteroid exposure | Hydrocortisone sodium succinate; Hydrocortisone sodium succinate 100 mg qd × 2 d; total HC-equivalent dose 200 mg |
| 26 | No | Severe | No corticosteroid exposure | No corticosteroid exposure |
| 27 | No | Severe | Hydrocortisone sodium succinate; Hydrocortisone sodium succinate 100 mg qd × 1 d; total HC-equivalent dose 100 mg; last dose before surgery 1 d; within 24 h: Yes | Hydrocortisone sodium succinate; Hydrocortisone sodium succinate 100 mg qd × 3 d; total HC-equivalent dose 300 mg |
| 28 | No | Severe | Hydrocortisone sodium succinate; Hydrocortisone sodium succinate 100 mg qd × 1 d; total HC-equivalent dose 100 mg; last dose before surgery 1 d; within 24 h: Yes | Hydrocortisone sodium succinate; Hydrocortisone sodium succinate 100 mg qd × 2 d; total HC-equivalent dose 200 mg |
| 29 | No | Severe | No corticosteroid exposure | No corticosteroid exposure |
| 30 | Yes | Severe | No corticosteroid exposure | No corticosteroid exposure |
| 31 | No | Severe | Hydrocortisone sodium succinate; Hydrocortisone sodium succinate 100 mg qd × 2 d; total HC-equivalent dose 200 mg; last dose before surgery 1 d; within 24 h: Yes | No corticosteroid exposure |
| 32 | Yes | Severe | No corticosteroid exposure | No corticosteroid exposure |
| 33 | Yes | Severe | Hydrocortisone sodium succinate; Hydrocortisone sodium succinate 100 mg qd × 1 d; total HC-equivalent dose 100 mg; last dose before surgery 1 d; within 24 h: Yes | Hydrocortisone sodium succinate; Hydrocortisone sodium succinate 100 mg qd × 1 d; total HC-equivalent dose 100 mg |
| 34 | Yes | Moderate | No corticosteroid exposure | No corticosteroid exposure |

HC: hydrocortisone. Patients without steroid exposure were assigned a cumulative dose of 0 mg.
