# Supplementary material for: Clinical trajectories after preoperative traditional Chinese medicine in biologic-refractory surgery- intended acute severe ulcerative colitis: an exploratory retrospective case series
Source: Front Immunol. 2026 Aug 24;17:1830986. doi: 10.3389/fimmu.2026.1830986 (PMC13547308; doi:10.3389/fimmu.2026.1830986)
Supplement: Supplementary file 3 [file Table2.docx]

**Supplementary Table S2A. Case-level TCM regimen summary and individualised modifications**

| **Case ID** | **Cohort** | **Core formula framework** | Individualised modifications | **Administration and safety** |
| --- | --- | --- | --- | --- |
| TCM-S01 | Surgical cohort | SLBZS + GGQLT modified | Haematochezia/haemostatic modification; Abdominal pain modification; Qi-stagnation modification; Purulent-bloody stool modification; Qi-deficiency modification | Decoction; Twice daily; 150–200 mL each time; 5–7 days; Prepared by the institutional pharmacy; adverse events: No TCM-related adverse events were documented in available records. |
| TCM-S02 | Surgical cohort | SLBZS + GGQLT modified | Haematochezia/haemostatic modification; Qi-stagnation modification; Purulent-bloody stool modification | Decoction; Twice daily; 150–200 mL each time; 5–7 days; Prepared by the institutional pharmacy; adverse events: No TCM-related adverse events were documented in available records. |
| TCM-S03 | Surgical cohort | SLBZS + GGQLT modified | Haematochezia/haemostatic modification; Qi-stagnation modification; Purulent-bloody stool modification; Qi-deficiency modification | Decoction; Twice daily; 150–200 mL each time; 5–7 days; Prepared by the institutional pharmacy; adverse events: No TCM-related adverse events were documented in available records. |
| TCM-S04 | Surgical cohort | SLBZS + GGQLT modified | Haematochezia/haemostatic modification; Purulent-bloody stool modification | Decoction; Twice daily; 150–200 mL each time; 5–7 days; Prepared by the institutional pharmacy; adverse events: No TCM-related adverse events were documented in available records. |
| TCM-S05 | Surgical cohort | SLBZS + GGQLT modified | Haematochezia/haemostatic modification; Abdominal pain modification; Purulent-bloody stool modification; Qi-deficiency modification | Decoction; Twice daily; 150–200 mL each time; 5–7 days; Prepared by the institutional pharmacy; adverse events: No TCM-related adverse events were documented in available records. |
| TCM-S06 | Surgical cohort | SLBZS + GGQLT modified | Haematochezia/haemostatic modification; Abdominal pain modification; Qi-stagnation modification; Purulent-bloody stool modification | Decoction; Twice daily; 150–200 mL each time; 5–7 days; Prepared by the institutional pharmacy; adverse events: No TCM-related adverse events were documented in available records. |
| TCM-R1 | Remission cohort | SLBZS + GGQLT modified | Haematochezia/haemostatic modification; Qi-stagnation modification; Poor spleen-stomach function modification; Purulent-bloody stool modification; Qi-deficiency modification | Decoction; Twice daily; 150–200 mL each time; 5–7 days; Prepared by the institutional pharmacy; adverse events: No TCM-related adverse events were documented in available records. |
| TCM-R2 | Remission cohort | SLBZS + GGQLT modified | Haematochezia/haemostatic modification; Abdominal pain modification; Purulent-bloody stool modification | Decoction; Twice daily; 150–200 mL each time; 5–7 days; Prepared by the institutional pharmacy; adverse events: No TCM-related adverse events were documented in available records. |
| TCM-R3 | Remission cohort | SLBZS + GGQLT modified | Haematochezia/haemostatic modification; Abdominal pain modification; Qi-stagnation modification; Purulent-bloody stool modification | Decoction; Twice daily; 150–200 mL each time; 5–7 days; Prepared by the institutional pharmacy; adverse events: No TCM-related adverse events were documented in available records. |
| TCM-R4 | Remission cohort | SLBZS + GGQLT modified | Haematochezia/haemostatic modification; Purulent-bloody stool modification | Decoction; Twice daily; 150–200 mL each time; 5–7 days; Prepared by the institutional pharmacy; adverse events: No TCM-related adverse events were documented in available records. |
| TCM-R5 | Remission cohort | SLBZS + GGQLT modified | Haematochezia/haemostatic modification; Poor spleen-stomach function modification; Purulent-bloody stool modification; Qi-deficiency modification | Decoction; Twice daily; 150–200 mL each time; 5–7 days; Prepared by the institutional pharmacy; adverse events: No TCM-related adverse events were documented in available records. |

TCM regimens were individualised based on SLBZS combined with GGQLT.

**Supplementary Table S2B. Detailed TCM prescriptions using academic English nomenclature**

| **Case ID** | **Cohort** | **Detailed prescription** |
| --- | --- | --- |
| TCM-S01 | Surgical cohort | Codonopsis Radix 15 g; Atractylodis Macrocephalae Rhizoma 12 g; Poria 15 g; Glycyrrhizae Radix et Rhizoma Praeparata cum Melle 6 g; Dioscoreae Rhizoma 30 g; Nelumbinis Semen 12 g; Lablab Semen Album 15 g; Coicis Semen 30 g; Amomi Fructus 3 g; Platycodonis Radix 6 g; Puerariae Lobatae Radix 30 g; Scutellariae Radix 9 g; Coptidis Rhizoma 3 g; Astragali Radix 15 g; Angelicae Sinensis Radix 9 g; Paeoniae Radix Alba 15 g; Agrimoniae Herba 18 g; Sanguisorbae Radix (raw) 12 g; Aucklandiae Radix 6 g. |
| TCM-S02 | Surgical cohort | Codonopsis Radix 12 g; Atractylodis Macrocephalae Rhizoma 12 g; Poria 15 g; Glycyrrhizae Radix et Rhizoma Praeparata cum Melle 6 g; Dioscoreae Rhizoma 30 g; Nelumbinis Semen 15 g; Lablab Semen Album 15 g; Coicis Semen 30 g; Amomi Fructus 3 g; Platycodonis Radix 6 g; Puerariae Lobatae Radix 30 g; Scutellariae Radix 9 g; Coptidis Rhizoma 3 g; Portulacae Herba 30 g; Pulsatillae Radix 12 g; Fraxini Cortex 9 g; Agrimoniae Herba 15 g; Sanguisorbae Radix (raw) 9 g; Aucklandiae Radix 6 g. |
| TCM-S03 | Surgical cohort | Codonopsis Radix 15 g; Atractylodis Macrocephalae Rhizoma 15 g; Poria 15 g; Glycyrrhizae Radix et Rhizoma Praeparata cum Melle 6 g; Dioscoreae Rhizoma 30 g; Nelumbinis Semen 15 g; Lablab Semen Album 20 g; Coicis Semen 20 g; Amomi Fructus 3 g; Platycodonis Radix 6 g; Puerariae Lobatae Radix 20 g; Scutellariae Radix 6 g; Coptidis Rhizoma 3 g; Astragali Radix 20 g; Euryales Semen 15 g; Mume Fructus 6 g; Agrimoniae Herba 15 g; Sanguisorbae Radix (raw) 9 g; Aucklandiae Radix 5 g. |
| TCM-S04 | Surgical cohort | Codonopsis Radix 9 g; Atractylodis Macrocephalae Rhizoma 9 g; Poria 12 g; Glycyrrhizae Radix et Rhizoma Praeparata cum Melle 6 g; Dioscoreae Rhizoma 20 g; Nelumbinis Semen 9 g; Lablab Semen Album 12 g; Coicis Semen 30 g; Amomi Fructus 3 g; Platycodonis Radix 6 g; Puerariae Lobatae Radix 30 g; Scutellariae Radix 12 g; Coptidis Rhizoma 3 g; Portulacae Herba 30 g; Pulsatillae Radix 15 g; Fraxini Cortex 9 g; Patriniae Herba 20 g; Sanguisorbae Radix (raw) 12 g; Agrimoniae Herba 15 g. |
| TCM-S05 | Surgical cohort | Codonopsis Radix 15 g; Atractylodis Macrocephalae Rhizoma 12 g; Poria 15 g; Glycyrrhizae Radix et Rhizoma Praeparata cum Melle 6 g; Dioscoreae Rhizoma 30 g; Nelumbinis Semen 12 g; Lablab Semen Album 15 g; Coicis Semen 30 g; Amomi Fructus 3 g; Platycodonis Radix 6 g; Puerariae Lobatae Radix 25 g; Scutellariae Radix 9 g; Coptidis Rhizoma 3 g; Astragali Radix 15 g; Angelicae Sinensis Radix 9 g; Salviae Miltiorrhizae Radix et Rhizoma 12 g; Sargentodoxae Caulis 30 g; Agrimoniae Herba 18 g; Sanguisorbae Radix (raw) 12 g. |
| TCM-S06 | Surgical cohort | Codonopsis Radix 12 g; Atractylodis Macrocephalae Rhizoma 12 g; Poria 15 g; Glycyrrhizae Radix et Rhizoma Praeparata cum Melle 6 g; Dioscoreae Rhizoma 24 g; Nelumbinis Semen 9 g; Lablab Semen Album 12 g; Coicis Semen 20 g; Amomi Fructus 3 g; Platycodonis Radix 6 g; Puerariae Lobatae Radix 20 g; Scutellariae Radix 9 g; Coptidis Rhizoma 3 g; Paeoniae Radix Alba 18 g; Aucklandiae Radix 6 g; Sophorae Flos Carbonisatus 12 g; Sanguisorbae Radix (raw) 12 g; Agrimoniae Herba 15 g; Sargentodoxae Caulis 20 g. |
| TCM-R1 | Remission cohort | Codonopsis Radix 15 g; Atractylodis Macrocephalae Rhizoma 15 g; Poria 15 g; Glycyrrhizae Radix et Rhizoma Praeparata cum Melle 6 g; Dioscoreae Rhizoma 30 g; Nelumbinis Semen 15 g; Lablab Semen Album 20 g; Coicis Semen 30 g; Amomi Fructus 3 g (decocted later); Platycodonis Radix 6 g; Puerariae Lobatae Radix 30 g; Scutellariae Radix 6 g; Coptidis Rhizoma 3 g; Euryales Semen 15 g; Mume Fructus 6 g; Agrimoniae Herba 15 g; Citri Reticulatae Pericarpium 6 g; Aucklandiae Radix 6 g; Massa Medicata Fermentata 12 g. |
| TCM-R2 | Remission cohort | Codonopsis Radix 12 g; Atractylodis Macrocephalae Rhizoma 12 g; Poria 15 g; Glycyrrhizae Radix et Rhizoma Praeparata cum Melle 6 g; Dioscoreae Rhizoma 30 g; Nelumbinis Semen 12 g; Lablab Semen Album 15 g; Coicis Semen 30 g; Amomi Fructus 3 g; Platycodonis Radix 6 g; Puerariae Lobatae Radix 25 g; Scutellariae Radix 9 g; Coptidis Rhizoma 6 g; Sanguisorbae Radix (raw) 12 g; Sophorae Flos Carbonisatus 12 g; Agrimoniae Herba 18 g; Bletillae Rhizoma 9 g; Angelicae Sinensis Radix 9 g; Paeoniae Radix Alba 15 g. |
| TCM-R3 | Remission cohort | Codonopsis Radix 12 g; Atractylodis Macrocephalae Rhizoma 12 g; Poria 15 g; Glycyrrhizae Radix et Rhizoma Praeparata cum Melle 6 g; Dioscoreae Rhizoma 24 g; Nelumbinis Semen 12 g; Lablab Semen Album 15 g; Coicis Semen 25 g; Amomi Fructus 3 g; Platycodonis Radix 6 g; Puerariae Lobatae Radix 30 g; Scutellariae Radix 9 g; Coptidis Rhizoma 3 g; Paeoniae Radix Alba 20 g; Aucklandiae Radix 6 g; Corydalis Rhizoma 9 g; Citri Sarcodactylis Fructus 9 g; Portulacae Herba 30 g; Agrimoniae Herba 15 g. |
| TCM-R4 | Remission cohort | Codonopsis Radix 9 g; Atractylodis Macrocephalae Rhizoma 9 g; Poria 12 g; Glycyrrhizae Radix et Rhizoma Praeparata cum Melle 6 g; Dioscoreae Rhizoma 20 g; Nelumbinis Semen 9 g; Lablab Semen Album 12 g; Coicis Semen 30 g; Amomi Fructus 3 g; Platycodonis Radix 6 g; Puerariae Lobatae Radix 30 g; Scutellariae Radix 12 g; Coptidis Rhizoma 3 g; Portulacae Herba 30 g; Pulsatillae Radix 15 g; Fraxini Cortex 9 g; Patriniae Herba 20 g; Sanguisorbae Radix (raw) 12 g; Sargentodoxae Caulis 30 g. |
| TCM-R5 | Remission cohort | Codonopsis Radix 15 g; Atractylodis Macrocephalae Rhizoma 15 g; Poria 15 g; Glycyrrhizae Radix et Rhizoma Praeparata cum Melle 6 g; Dioscoreae Rhizoma 30 g; Nelumbinis Semen 15 g; Lablab Semen Album 20 g; Coicis Semen 20 g; Amomi Fructus 3 g; Platycodonis Radix 6 g; Puerariae Lobatae Radix 20 g; Scutellariae Radix 6 g; Coptidis Rhizoma 3 g; Astragali Radix 20 g; Pseudostellariae Radix 15 g; Euryales Semen 15 g; Mume Fructus 6 g; Agrimoniae Herba 15 g; Citri Reticulatae Pericarpium 6 g. |

Detailed prescriptions are presented using academic English nomencl
