# Supplementary material for: Clinical trajectories after preoperative traditional Chinese medicine in biologic-refractory surgery- intended acute severe ulcerative colitis: an exploratory retrospective case series
Source: Front Immunol. 2026 Aug 24;17:1830986. doi: 10.3389/fimmu.2026.1830986 (PMC13547308; doi:10.3389/fimmu.2026.1830986)
Supplement: Supplementary file 4 [file Table3.docx]

Supplementary Table S3. Documented prior biologic and rescue-treatment information in patients with retrievable records

Only records with available biologic-agent or rescue-treatment information are shown. Treatment line could not be reconstructed consistently; the table therefore reports the documented number of administrations when available. Detailed duration, efficacy reassessment timepoints, trough concentrations, and anti-drug antibody results were not routinely available and were not imputed. No JAK-inhibitor rescue exposure was identified. A dash indicates unavailable information.

| **Case ID** | **Cohort** | **Surgery status** | **Documented agent/rescue therapy** | No. of administrations | **Documented exposure details** | **Documented failure/reason for referral** |
| --- | --- | --- | --- | --- | --- | --- |
| Case 6 | Non-TCM | Underwent surgery | Cyclosporine rescue | - | Cyclosporine rescue documented | - |
| Case 8 | Non-TCM | Underwent surgery | Infliximab | 3 | Started 2019-04-01 | Intolerance/adverse event |
| Case 8 | Non-TCM | Underwent surgery | Vedolizumab | 2 | - | Recurrent haematochezia (>10 stools/day) with fever up to 38 °C |
| Case 11 | Non-TCM | Underwent surgery | Biologic agent unspecified in the medical record | 1 | Started 2018-05-01 | Intolerance/adverse event |
| Case 11 | Non-TCM | Underwent surgery | Infliximab | - | Started 2019-06-18 | Inadequate clinical response |
| Case 14 | Non-TCM | Underwent surgery | Vedolizumab (agent name inconsistently recorded) | - | - | Inadequate clinical response |
| Case 14 | Non-TCM | Underwent surgery | Infliximab | 7 | 300 mg IV; started 2020-02-06; last dose 2020-08-25 | Inadequate clinical response |
| Case 17 | Non-TCM | Underwent surgery | Infliximab | 3 | - | Inadequate clinical response |
| Case 18 | Non-TCM | Underwent surgery | Infliximab | 2 | 300 mg; started 2020-02-05; last dose 2020-02-19 | Inadequate clinical response |
| Case 18 | Non-TCM | Underwent surgery | Cyclosporine rescue | - | Cyclosporine rescue documented | - |
| Case 20 | Non-TCM | Underwent surgery | Infliximab | 3 | 500 mg; started 2022-02-22; last dose 2022-03-28 | Inadequate clinical response |
| Case 26 | Non-TCM | Underwent surgery | Infliximab | 5 | 300 mg; started 2021-09-07 | Rash with suspected infliximab hypersensitivity |
| Case 26 | Non-TCM | Underwent surgery | Vedolizumab | 5 | 300 mg; started 2022-03-17; last dose 2022-08-12 | - |
| Case 22 | TCM | Underwent surgery | Vedolizumab | 3 | Started 2021-08-01 | Inadequate clinical response |
| Case 22 | TCM | Underwent surgery | Infliximab | 6 | Started 2021-10-10; last dose 2022-03-09 | Inadequate clinical response |
| Case 32 | TCM | Underwent surgery | Infliximab | 1 | 400 mg; administered 2024-07-05 | Inadequate clinical response after infliximab rescue; patient preference supported surgery |
| Case 33 | TCM | Underwent surgery | Vedolizumab | 6 | - | Inadequate clinical response |
| Case 35 | TCM | Surgery cancelled | Infliximab | 3 | 300 mg; started 2023-03-07; last dose 2023-04-20 | Inadequate clinical response |
| Case 37 | TCM | Surgery cancelled | Infliximab | 2 | 300 mg; started 2025-04-24; last dose 2025-04-28 | Inadequate clinical response |
| Case 39 | TCM | Surgery cancelled | Infliximab | 2 | 400 mg; started 2025-04-23 | Inadequate clinical response |
