# Supplementary material for: Clinical trajectories after preoperative traditional Chinese medicine in biologic-refractory surgery- intended acute severe ulcerative colitis: an exploratory retrospective case series
Source: Front Immunol. 2026 Aug 24;17:1830986. doi: 10.3389/fimmu.2026.1830986 (PMC13547308; doi:10.3389/fimmu.2026.1830986)
Supplement: Supplementary file 5 [file Table4.docx]

**Supplementary Table S4. Case-level perioperative concomitant treatments and supportive care**

Cumulative quantities were abstracted from available clinical records. The precise aggregation period was not uniformly documented.

**Part A. Antibiotic exposure and nutritional support in the 34 surgical patients**

| **Case ID** | **Group** | **Surgery date** | **Approach** | **Scope** | **Antibiotic agents** | **Antibiotic duration, d** | **Enteral nutrition** | **Parenteral nutrition** | **PN duration, d** |
| --- | --- | --- | --- | --- | --- | --- | --- | --- | --- |
| Case 1 | Non-TCM | 2024-12-27 | Laparoscopic | total colectomy | Metronidazole; Ceftizoxime sodium; Cefradine capsules | 71 | No | Yes | 34 |
| Case 2 | Non-TCM | 2021-05-21 | Laparoscopic | IPAA | Ceftizoxime sodium; Ornidazole | 16 | No | Yes | 14 |
| Case 3 | Non-TCM | 2020-07-30 | Laparoscopic | total colectomy | Ceftizoxime sodium; Ornidazole | 15 | No | Yes | 21 |
| Case 4 | Non-TCM | 2020-09-07 | Laparoscopic | total colectomy | Ceftizoxime sodium; Ornidazole | 21 | No | Yes | 20 |
| Case 5 | Non-TCM | 2021-09-25 | Laparoscopic | IPAA | Ceftriaxone sodium; Metronidazole; Ornidazole; Ceftizoxime sodium; Levofloxacin | 15 | No | Yes | 19 |
| Case 6 | Non-TCM | 2020-10-19 | Laparoscopic | total colectomy | Metronidazole; Ceftizoxime sodium; Imipenem | 15 | No | Yes | 16 |
| Case 7 | Non-TCM | 2022-12-04 | Laparoscopic | IPAA | Metronidazole; Levofloxacin; Cefoxitin sodium | 17 | No | Yes | 16 |
| Case 8 | Non-TCM | 2020-09-30 | Laparoscopic | IPAA | Levofloxacin; Metronidazole; Ornidazole | 18 | No | Yes | 17 |
| Case 9 | Non-TCM | 2024-06-30 | Laparoscopic | IPAA | Ceftizoxime sodium; Levofloxacin | 13 | No | Yes | 12 |
| Case 10 | Non-TCM | 2024-06-09 | Laparoscopic | IPAA | Ceftizoxime sodium; Levofloxacin | 8 | No | Yes | 14 |
| Case 11 | Non-TCM | 2020-06-28 | Laparoscopic | IPAA | Ceftizoxime sodium; Levofloxacin | 8 | Yes | Yes | 3 |
| Case 12 | Non-TCM | 2020-07-14 | Laparoscopic | IPAA | Ceftizoxime sodium; Levofloxacin; Mezlocillin sodium/sulbactam sodium | 14 | Yes | Yes | 25 |
| Case 13 | Non-TCM | 2020-08-17 | Laparoscopic | total colectomy | Mezlocillin sodium/sulbactam sodium; Meropenem; Cefamandole nafate | 23 | No | Yes | 11 |
| Case 14 | Non-TCM | 2020-09-10 | Laparoscopic | IPAA | Ceftizoxime sodium; Levofloxacin; Cefradine | 5 | No | Yes | 11 |
| Case 15 | Non-TCM | 2020-12-18 | Laparoscopic | IPAA | Mezlocillin sodium/sulbactam sodium | 16 | No | Yes | 18 |
| Case 16 | Non-TCM | 2021-01-12 | Laparoscopic | IPAA | Mezlocillin sodium/sulbactam sodium; Levofloxacin | 11 | No | Yes | 13 |
| Case 17 | Non-TCM | 2021-04-26 | Laparoscopic | IPAA | Levofloxacin; Cefoperazone sodium/sulbactam sodium; Mezlocillin sodium/sulbactam sodium | 20 | No | Yes | 21 |
| Case 18 | Non-TCM | 2021-03-29 | Laparoscopic | IPAA | Piperacillin sodium/tazobactam sodium; Levofloxacin; Ornidazole; Mezlocillin sodium/sulbactam sodium; Cefdinir | 37 | No | Yes | 36 |
| Case 19 | Non-TCM | 2022-03-31 | Laparoscopic | IPAA | Levofloxacin; Ornidazole; Ceftizoxime sodium | 26 | No | Yes | 20 |
| Case 20 | Non-TCM | 2022-05-05 | Laparoscopic | subtotal colectomy | Ornidazole; Ceftizoxime sodium; Meropenem | 30 | No | Yes | 29 |
| Case 21 | Non-TCM | 2022-05-26 | Laparoscopic | IPAA | Levofloxacin; Ornidazole; Ceftizoxime sodium | 35 | No | Yes | 11 |
| Case 22 | TCM | 2022-06-23 | Laparoscopic | subtotal colectomy | Levofloxacin; Ornidazole; Ceftizoxime sodium | 20 | No | Yes | 14 |
| Case 23 | TCM | 2022-07-26 | Laparoscopic | subtotal colectomy | Vancomycin; Ceftizoxime sodium | 32 | No | Yes | 26 |
| Case 24 | Non-TCM | 2023-01-03 | Laparoscopic | subtotal colectomy | Ornidazole; Ceftizoxime sodium | 15 | No | Yes | 17 |
| Case 25 | Non-TCM | 2023-03-07 | Laparoscopic | IPAA | Ornidazole; Ceftizoxime sodium | 11 | No | Yes | 11 |
| Case 26 | Non-TCM | 2023-03-27 | Laparoscopic | IPAA | Ceftizoxime sodium; Levofloxacin | 19 | No | Yes | 18 |
| Case 27 | Non-TCM | 2023-08-14 | Laparoscopic | subtotal colectomy | Ornidazole; Ceftizoxime sodium | 17 | No | Yes | 17 |
| Case 28 | Non-TCM | 2023-04-26 | Laparoscopic | subtotal colectomy | Levofloxacin; Ornidazole; Ceftizoxime sodium | 22 | No | Yes | 14 |
| Case 29 | Non-TCM | 2023-06-12 | Open | subtotal colectomy | Ceftriaxone sodium; Ornidazole | 38 | No | Yes | 28 |
| Case 30 | TCM | 2023-08-15 | Laparoscopic | total colectomy | Ornidazole; Cefmetazole sodium | 8 | No | Yes | 17 |
| Case 31 | Non-TCM | 2023-08-07 | Laparoscopic | total colectomy | Levofloxacin; Ornidazole; Metronidazole; Cefmetazole sodium | 35 | Yes | Yes | 14 |
| Case 32 | TCM | 2024-07-28 | Laparoscopic | IPAA | Cefoperazone sodium/sulbactam sodium; Metronidazole; Meropenem; Mezlocillin sodium/sulbactam sodium; Imipenem/cilastatin sodium | 38 | No | Yes | 52 |
| Case 33 | TCM | 2025-05-29 | Laparoscopic | subtotal colectomy | Mezlocillin sodium/sulbactam sodium; Cefmetazole sodium | 8 | No | Yes | 16 |
| Case 34 | TCM | 2021-03-08 | Open | subtotal colectomy | Mezlocillin sodium/sulbactam sodium | 10 | No | Yes | 31 |

**Part B. Additional perioperative supportive care in the 34 surgical patients**

| **Case ID** | **Group** | **Albumin, g** | **RBC, U** | **Plasma, mL** | **IV iron, mg** | **Anticoagulation** | Antidiarrhoeal agents | **ERAS** | **Infection control** | **Bowel preparation** |
| --- | --- | --- | --- | --- | --- | --- | --- | --- | --- | --- |
| Case 1 | Non-TCM | 315 | 4 | 0 | Not documented | Yes | No | Not documented | Yes | Yes |
| Case 2 | Non-TCM | 50 | 0 | 0 | Not documented | No | Yes | Not documented | Yes | Yes |
| Case 3 | Non-TCM | 195 | 1.5 | 400 | Not documented | No | No | Not documented | Yes | Yes |
| Case 4 | Non-TCM | 145 | 4 | 300 | Not documented | No | No | Not documented | Yes | Yes |
| Case 5 | Non-TCM | 180 | 1.5 | 200 | Not documented | No | No | Not documented | Yes | Yes |
| Case 6 | Non-TCM | 100 | 2 | 150 | Not documented | No | No | Not documented | Yes | Yes |
| Case 7 | Non-TCM | 135 | 0 | 0 | Not documented | No | Yes | Not documented | Yes | Yes |
| Case 8 | Non-TCM | 120 | 3 | 200 | Not documented | No | No | Not documented | Yes | Yes |
| Case 9 | Non-TCM | 0 | 0 | 0 | Not documented | No | No | Not documented | Yes | Yes |
| Case 10 | Non-TCM | 50 | 0 | 0 | Not documented | No | No | Not documented | Yes | Yes |
| Case 11 | Non-TCM | 20 | 0 | 0 | Not documented | No | No | Not documented | Yes | Yes |
| Case 12 | Non-TCM | 100 | 0 | 0 | Not documented | No | No | Not documented | Yes | Yes |
| Case 13 | Non-TCM | 120 | 5 | 600 | Not documented | Yes | No | Not documented | Yes | Yes |
| Case 14 | Non-TCM | 0 | 0 | 0 | Not documented | No | No | Not documented | Yes | Yes |
| Case 15 | Non-TCM | 50 | 0 | 0 | Not documented | No | No | Not documented | Yes | Yes |
| Case 16 | Non-TCM | 20 | 0 | 0 | Not documented | No | No | Not documented | Yes | Yes |
| Case 17 | Non-TCM | 20 | 0 | 0 | Not documented | No | No | Not documented | Yes | Yes |
| Case 18 | Non-TCM | 225 | 3 | 250 | Not documented | Yes | Yes | Not documented | Yes | Yes |
| Case 19 | Non-TCM | 160 | 0 | 0 | Not documented | No | No | Not documented | Yes | Yes |
| Case 20 | Non-TCM | 110 | 0 | 0 | Not documented | Yes | Yes | Not documented | Yes | Yes |
| Case 21 | Non-TCM | 60 | 0 | 0 | Not documented | Yes | No | Not documented | Yes | Yes |
| Case 22 | TCM | 85 | 3 | 400 | Not documented | Yes | No | Not documented | Yes | Yes |
| Case 23 | TCM | 145 | 3 | 425 | Not documented | No | No | Not documented | Yes | Yes |
| Case 24 | Non-TCM | 115 | 2 | 500 | Not documented | No | No | Not documented | Yes | Yes |
| Case 25 | Non-TCM | 45 | 0 | 0 | Not documented | No | No | Not documented | Yes | Yes |
| Case 26 | Non-TCM | 65 | 0 | 0 | Not documented | Yes | No | Not documented | Yes | Yes |
| Case 27 | Non-TCM | 40 | 1.5 | 375 | Not documented | No | No | Not documented | Yes | Yes |
| Case 28 | Non-TCM | 10 | 0 | 0 | Not documented | No | No | Not documented | Yes | Yes |
| Case 29 | Non-TCM | 90 | 7 | 1150 | Not documented | No | No | Not documented | Yes | Yes |
| Case 30 | TCM | 150 | 0 | 0 | Not documented | No | No | Not documented | Yes | Yes |
| Case 31 | Non-TCM | 40 | 0 | 0 | Not documented | Yes | No | Not documented | Yes | Yes |
| Case 32 | TCM | 150 | 10.5 | 1200 | 900 | No | No | Not documented | Yes | Yes |
| Case 33 | TCM | 60 | 2 | 600 | Not documented | No | No | Not documented | Yes | Yes |
| Case 34 | TCM | 0 | 0 | 100 | Not documented | No | No | Not documented | Yes | Yes |

ERAS, enhanced recovery after surgery; IV, intravenous; PN, parenteral nutrition; RBC, red blood cell.

**Part C. Available supportive-care information in the five surgery-cancelled remission cases**

| **Case ID** | **Group** | **Surgery status** | **Antibiotics** | **Antibiotic agents** | **Parenteral nutrition** | **Other supportive-care data** |
| --- | --- | --- | --- | --- | --- | --- |
| TCM-R1 | TCM | Surgery cancelled / not performed | Yes | Levofloxacin; Ornidazole; Ceftizoxime sodium | Yes | Not retrievable from available clinical records. |
| TCM-R2 | TCM | Surgery cancelled / not performed | Yes | Ceftizoxime sodium; Levofloxacin | Yes | Not retrievable from available clinical records. |
| TCM-R3 | TCM | Surgery cancelled / not performed | Yes | Cefoperazone sodium/sulbactam sodium | Yes | Not retrievable from available clinical records. |
| TCM-R4 | TCM | Surgery cancelled / not performed | Yes | Mezlocillin sodium/sulbactam sodium; Levofloxacin | Yes | Not retrievable from available clinical records. |
| TCM-R5 | TCM | Surgery cancelled / not performed | Yes | Ornidazole; Ceftizoxime sodium | Yes | Not retrievable from available clinical records. |

Postoperative variables were not applicable to the five patients whose surgery was cancelled.

Supplementary Table S5. Results of infection screening, including Clostridioides difficile, stool pathogens, cytomegalovirus testing, urine culture, and blood culture.

Infection-screening results were classified as positive/abnormal, negative, or indeterminate from the clinical records. Negative results were reported as negative. Data on pathogen-directed treatment were not consistently available; antibiotic exposure and documented infection-control treatment are reported in Supplementary Table S4. Infection control before surgery was explicitly recorded in six patients.

| Infection assessment | Positive/abnormal, n | Negative, n | Other/indeterminate, n | Total, N | Management | Comment |
| --- | --- | --- | --- | --- | --- | --- |
| C. difficile toxin/PCR | 1 | 38 | 0 | 39 | Unavailable | — |
| Stool culture/pathogen | 0 | 38 | 1 | 39 | Not applicable | One indeterminate result reported spores without organism specification |
| CMV blood PCR | 6 | 33 | 0 | 39 | Unavailable | — |
| CMV immunohistochemistry | 2 | 37 | 0 | 39 | Unavailable | — |
| CMV tissue PCR | 0 | 39 | 0 | 39 | Not applicable | — |
| Other enteric pathogen testing | 0 | 38 | 1 | 39 | Unavailable | One indeterminate colonoscopic impression suggested possible CMV |
| Urine culture/pathogen | 0 | 39 | 0 | 39 | Not applicable | — |
| Blood culture/pathogen | 0 | 39 | 0 | 39 | Not applicable | — |

Supplementary Figure S1. Individual clinical timelines of the 11 TCM-treated, surgery-intended patients with acute severe ulcerative colitis. Admission was defined as day 0. Infliximab rescue therapy and TCM are shown as dated exposure intervals. Corticosteroid regimens, antibiotic exposure, and parenteral nutrition are shown as annotations because exact start and stop dates were not uniformly documented. The endpoint denotes surgery or the documented date of surgery cancellation. ASUC, acute severe ulcerative colitis; IFX, infliximab; TCM, traditional Chinese medicine; PN, parenteral nutrition; ABx, antibiotics; HC, hydrocortisone; MPS, methylprednisolone sodium succinate.

Supplementary Figure S2. Paired endoscopic images of the five remission cases. Panels a–e correspond to TCM-R1 to TCM-R5, respectively. Each panel shows pre-treatment and post-treatment endoscopic images. Intestinal segment labels are presented in English. Pre-treatment MES was 3 in all five cases; post-treatment MES was 1, 0, 1, 1, and 1 in TCM-R1 to TCM-R5, respectively. The TCM-R2 post-treatment panel displays the image set corresponding to the independently assessed MES 0 examination. Comparable or near-comparable bowel segments were used where available. Images were independently reviewed in a blinded manner by two endoscopists. Exact post-treatment examination dates could not be reliably linked to every archived image set; the latest documented endoscopic follow-up month is shown in Supplementary Figure S1. These five selected patients experienced remission after integrative management including TCM and did not undergo surgery.
