# Supplementary material for: Clinical trajectories after preoperative traditional Chinese medicine in biologic-refractory surgery- intended acute severe ulcerative colitis: an exploratory retrospective case series
Source: Front Immunol. 2026 Aug 24;17:1830986. doi: 10.3389/fimmu.2026.1830986 (PMC13547308; doi:10.3389/fimmu.2026.1830986)
Supplement: Supplementary file 6 [file Table5.docx]

Supplementary Table S5. Results of infection screening, including Clostridioides difficile, stool pathogens, cytomegalovirus testing, urine culture, and blood culture.

Infection-screening results were classified as positive/abnormal, negative, or indeterminate from the clinical records. Negative results were reported as negative. Data on pathogen-directed treatment were not consistently available; antibiotic exposure and documented infection-control treatment are reported in Supplementary Table S4. Infection control before surgery was explicitly recorded in six patients.

| Infection assessment | Positive/abnormal, n | Negative, n | Other/indeterminate, n | Total, N | Management | Comment |
| --- | --- | --- | --- | --- | --- | --- |
| C. difficile toxin/PCR | 1 | 38 | 0 | 39 | Unavailable | — |
| Stool culture/pathogen | 0 | 38 | 1 | 39 | Not applicable | One indeterminate result reported spores without organism specification |
| CMV blood PCR | 6 | 33 | 0 | 39 | Unavailable | — |
| CMV immunohistochemistry | 2 | 37 | 0 | 39 | Unavailable | — |
| CMV tissue PCR | 0 | 39 | 0 | 39 | Not applicable | — |
| Other enteric pathogen testing | 0 | 38 | 1 | 39 | Unavailable | One indeterminate colonoscopic impression suggested possible CMV |
| Urine culture/pathogen | 0 | 39 | 0 | 39 | Not applicable | — |
| Blood culture/pathogen | 0 | 39 | 0 | 39 | Not applicable | — |

Supplementary Figure S1. Individual clinical timelines of the 11 TCM-treated, surgery-intended patients with acute severe ulcerative colitis. Admission was defined as day 0. Infliximab rescue therapy and TCM are shown as dated exposure intervals. Corticosteroid regimens, antibiotic exposure, and parenteral nutrition are shown as annotations because exact start and stop dates were not uniformly documented. The endpoint denotes surgery or the documented date of surgery cancellation. ASUC, acute severe ulcerative colitis; IFX, infliximab; TCM, traditional Chinese medicine; PN, parenteral nutrition; ABx, antibiotics; HC, hydrocortisone; MPS, methylprednisolone sodium succinate.

Supplementary Figure S2. Paired endoscopic images of the five remission cases. Panels a–e correspond to TCM-R1 to TCM-R5, respectively. Each panel shows pre-treatment and post-treatment endoscopic images. Intestinal segment labels are presented in English. Pre-treatment MES was 3 in all five cases; post-treatment MES was 1, 0, 1, 1, and 1 in TCM-R1 to TCM-R5, respectively. The TCM-R2 post-treatment panel displays the image set corresponding to the independently assessed MES 0 examination. Comparable or near-comparable bowel segments were used where available. Images were independently reviewed in a blinded manner by two endoscopists. Exact post-treatment examination dates could not be reliably linked to every archived image set; the latest documented endoscopic follow-up month is shown in Supplementary Figure S1. These five selected patients experienced remission after integrative management including TCM and did not undergo surgery.
